# NUF2 and NEK2 promote malignant progression of gallbladder cancer by remodeling the extracellular matrix

**DOI:** 10.1093/carcin/bgaf019

**Published:** 2025-04-12

**Authors:** Ming Gao, Peng Ye, Yutong Zhang, Yarong Guo, Jun Xu

**Affiliations:** Hepatobiliary and Pancreatic Surgery and Liver Transplantation Center, First Hospital of Shanxi Medical University, No. 85 Jiefang South Road, Taiyuan, Shanxi 030001, China; Faculty of Graduate Studies, Shanxi Medical University, No. 56 Xinjian South Road, Taiyuan, Shanxi 030001, China; Faculty of Graduate Studies, Shanxi Medical University, No. 56 Xinjian South Road, Taiyuan, Shanxi 030001, China; Faculty of Graduate Studies, Shanxi Medical University, No. 56 Xinjian South Road, Taiyuan, Shanxi 030001, China; Key Laboratory of Cellular Physiology of the Ministry of Education (Shanxi Medical University), Translational Medicine Research Center, Department of Pathology, Shanxi Medical University, No. 56 Xinjian South Road, Taiyuan, Shanxi 030001, China; Oncology Department, Shanxi Bethune Hospital, No. 99 Longcheng Street, Taiyuan, Shanxi 030032, China; Hepatobiliary and Pancreatic Surgery and Liver Transplantation Center, First Hospital of Shanxi Medical University, No. 85 Jiefang South Road, Taiyuan, Shanxi 030001, China

**Keywords:** bioinformatics, extracellular matrix, gallbladder cancer, proliferation, stiffened matrix

## Abstract

Gallbladder cancer (GBC) ranks as the most common malignant tumor of the biliary tract, which has been characterized by late diagnosis, low excisional rate, and poor prognosis. Recent studies exploring the roles of malignant progression-associated genes in GBC remain limited. Our study aims to identify significant hub genes involved in its pathogenesis, which may serve as novel potential therapeutic targets for GBC. Here, we employed RNA-seq analysis to identify differentially expressed genes (DEGs) of seven GBC samples and five matched adjacent samples. After screening the DEGs in clinical sequencing data and GSE139682, we further obtained 549 genes with consistent expression trends in two datasets, including 155 upregulated and 394 downregulated genes. Gene Ontology (GO) enrichment analysis revealed that these genes were significantly enriched in extracellular matrix (ECM)-related processes, such as organization, structure, and composition, which hint to us that remodeling of ECM may be the main driving factor for the malignant progression of GBC. In addition, we screened 17 candidate hub genes through protein-protein interaction (PPI) network analysis and Cytoscape, subsequent GO and Kyoto Encyclopedia of Genes and Genomes (KEGG) enrichment analyses showed that the remodeled ECM mainly functions by affecting cell division. Moreover, we found that NEK2 and NUF2 were overexpressed in GBC tumor tissues and validated their function in the pro-proliferation of GBC cells. Our results highlight that NEK2 and NUF2 may be hub genes promoting the malignant progression of GBC and are expected to be reliable new therapeutic targets for GBC.

## Graphical Abstract

**Graphical Abstract:**
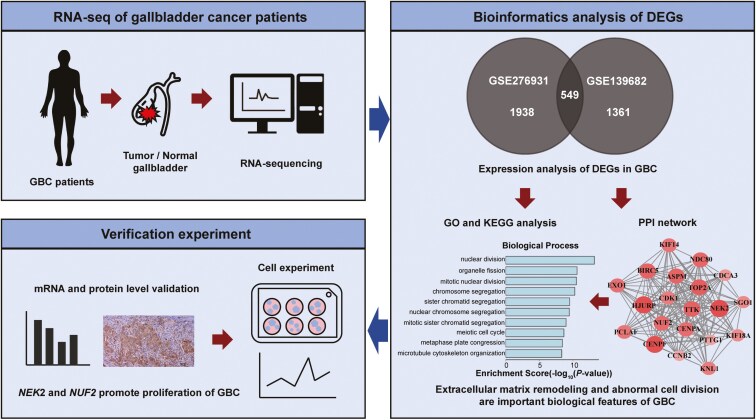

## Introduction

Gallbladder cancer (GBC) is the most common cancer of the biliary tract. In 2022, approximately 122 462 new cases of GBC were reported in the world, resulting in 89 031 deaths (1). The incidence of GBC increases with age and exhibits significant geographical variation (2,3). According to the 8th American Joint Committee on Cancer (AJCC) guidelines, surgical resection is the most effective treatment for early GBC, while for advanced patients, adjuvant therapy can provide more benefits to them (4). As it rarely exhibits early symptoms, screening and early detection at a curable stage remain challenging. As a result, the majority of GBC patients are diagnosed at the advanced stages and miss the opportunity for surgical treatment. Notably, GBC is characterized by inherent resistance to chemotherapy, resulting in a very poor prognosis for patients (2). With the exploration of therapeutic targets, such as FGFR and IDH1, targeted therapies have made important progress in the treatment of biliary tract cancer (BTC) (5–8). Although GBC is one type of BTC, these targets are rare in GBC. Therefore, identifying new targets for GBC may provide better prospects for targeted treatment of GBC.

With the widespread adoption of RNA sequencing (RNA-seq), the discovery of new targets for tumor treatment has become more convenient (9). The pivotal link is the combination of RNA-seq and bioinformatics analysis, which can help to find molecular targets and analyze the mechanism of tumorigenesis at the molecular level. Nowadays, this technology has been extensively utilized for identifying therapeutic targets in cancer, such as breast cancer, gastric cancer, and hepatocellular carcinoma (10–12). Nevertheless, there is relatively little research on gallbladder cancer.

In this study, we obtained mRNA expression profiles of gallbladder cancer tissues and paired adjacent cancerous tissues using the RNA-seq analysis. Then, we combined transcriptome data of GSE139682 from the Gene Expression Omnibus (GEO) database (13) to screen out the intersection of differentially expressed genes (DEGs). Finally, we conducted a bioinformatic analysis to identify the hub genes, and further experimental validation was performed to confirm their roles. This study aimed to identify potential therapeutic targets for the diagnosis and treatment of GBC.

## Methods

### Patients and sample processing

We recruited seven GBC patients and obtained their cancerous and adjacent tissues, who underwent radical resection of gallbladder cancer at the First Hospital of Shanxi Medical University. All patients were diagnosed with gallbladder adenocarcinoma through pathological examination. After resection, specimens were immediately frozen in liquid nitrogen and stored at −80°C for long-term storage. Finally, we performed transcriptome sequencing on the specimens. This study was approved by the Ethics Committee of the First Hospital of Shanxi Medical University, and informed consent was obtained from each participant.

### RNA-seq analysis and data processing

Total RNA was extracted by TRIzol (Mei5bio, MF034-01), according to the manufacturer’s instructions, and DNase I was used to eliminate the DNA contamination. The integrality of RNA was detected by Agilent 2100 bioanalyzer, and two poor-quality adjacent cancer tissues were excluded. Then, only qualified total RNA was used for RNA-seq library construction by NEBNext® Ultra II RNA Library Prep Kit for Illumina. Finally, they were used for subsequent sequencing experiments on the NovaSeq 6000 platform (Illumina). The raw data in FASTQ format were processed to obtain clean data by removing low-quality Reads and Reads containing adapters. Compare paired-end readings with the reference genome (Hg38) using Hisat2 v2.0.5. HTSeq (0.9.1) was used to calculate the number of reads mapped to each gene, and then we used FPKM to standardize the expression.

### Public databases

The gene expression dataset GSE139682, based on the GPL20795 platform was obtained from the GEO database. The GSE139682 dataset contains 20 clinical samples, including 10 gallbladder cancer (GBC) and 10 paired adjacent tissues.

### DEG identification

Difference expression genes between seven GBC samples and five matched adjacent samples were analyzed using the DESeq2 R package (version 1.30.0). Significant *P*-value < 0.05 and expression difference multiple |log2FoldChange| > 1 were identified as the thresholds for the screening of DEGs. Then, overlapping DEGs among the GSE139682 and clinical sequencing data were found through a Venn diagram.

### Gene Ontology (GO) enrichment and Kyoto Encyclopedia of Genes and Genomes (KEGG) pathway analyses

DEGs were subjected to the KEGG Orthology-Based Annotation System (KOBAS, http://bioinfo.org/kobas) to interpret the GO functions and KEGG pathways (14). Among these, GO function analysis contains a biological process (BP), cellular component (CC), and molecular function (MF). Finally, the results were visualized using the Bioinformatics (www.bioinformatics.com) free online platform.

### Protein-protein interaction (PPI) network and modules analysis

The DEG interactions were analyzed using the online analysis tool STRING (http://string-db.org), with a confidence of 0.7. The results of protein-protein interaction (PPI) interactions were visualized by Cytoscape software (version 3.10.1). In addition, the MCODE plugin in Cytoscape software was used for identifying the pivotal modules in the PPI network. The screening criteria were set as follows: MCODE score > 5, degree-cuﬀ = 2, node score = 0.2, max depth = 100, and *K* score = 2. And Cytoscape plugin CytoHubba was applied to identify key genes via three analysis methods: maximal clique centrality (MCC), maximum neighborhood component (MNC), and degree.

### Expression levels and survival analysis of candidate hub genes in TCGA

The gene expression profiling and interactive analysis database (GEPIA, http://gepia.cancer-pku.cn) contained gene expression of 36 cholangiocarcinoma samples and nine non-tumor samples. The expression levels of hub genes were analyzed using GEPIA.

### qRT-PCR

Total RNA was extracted using TRIzol reagent (Mei5bio, MF034-01). Subsequently, we used PrimeScript™ RT Reagent Kit (Takara, RR047Q) to transcribe 1 µg of the total RNA into cDNA templates. And then, qRT-PCR assays were performed on the StepOnePlus® Real-Time PCR system (ThermoFisher, USA), using 2X M5 HiPer Dual SYBRgreen Realtime PCR Super mix (Mei5bio, MF013). *GAPDH* was used as the standardized internal reference. The relative gene expression was calculated by the 2^(−Δ*CT*)^ method. The sequences of PCR primers are listed in Supplementary Table S1.

### Western blotting

Cells were lysed in RIPA lysis buffer (Beyotime, P0013B) with protease inhibitors (MCE, HY-K0010). A total of 10%–15% SDS-PAGE was used to separate protein products, and the protein products were transferred to the PVDF membrane. The protein bands were visualized under the ChemiDoc MP Imaging System (BIO-RAD, USA). Primary antibodies used were listed as follows: antibodies against NUF2 (1:200, Proteintech, 15731-1-AP), NEK2 (1:200, Proteintech, 24171-1-AP), and GAPDH (Proteintech, 10494-1-AP).

### Immunohistochemical (IHC) staining and hematoxylin and eosin (H&E) staining

After fixing with paraformaldehyde and dehydrating with ethanol, the tissues were embedded in paraffin. Subsequently, they were cut into 2.5 μm slices and stained with H&E. For IHC staining, we incubate the slices with primary antibodies targeting NUF2 (1:200, Proteintech, 15731-1-AP), KIF14 (1:200, Proteintech, 26000-1-AP), NEK2 (1:200, Proteintech, 24171-1-AP), and Ki67 (1:5000, Proteintech, 28074-1-AP) overnight at 4°C. Then, they were incubated with rabbit/mouse second antibody at 37°C for 1 h. Chromogen development was accomplished with diaminobenzidine (DAB), and images were obtained by Nikon microscope.

### Cell culture and treatment

Human GBC cell lines GBC-SD and NOZ were purchased from iCell Bioscience Inc Company (Shanghai, China). Cell lines were subjected to identity authentication using a short tandem repeat profiling method. NOZ cells were cultured in a DMEM medium. GBC-SD cells were cultured in RPMI-1640 medium. All media were supplemented with 10% fetal bovine serum and all cells were cultured at 37°C in a humidified incubator containing 5% CO_2_. Small interfering RNA (siRNA) specifically targeting NUF2 and NEK2 were obtained from RiboBio (Guangzhou, China) and transfected using Lipofectamine 2000 (Mei5bio, MF135-01). The GBC-SD and NOZ cells were plated onto six-well plates, and transfected using siRNA when they reached a confluency of 30%–40%. After 6 h of transfection, the transfection medium was replaced with a fresh medium. Cell colony formation and cell viability assays were conducted 48 h after transfection. The siRNA sequences are listed in Supplementary Table S2.

### Cell colony formation assay and cell viability assay

Totally 1 × 10^3^ cells were seeded into six-well plates. After incubation for 10 days, cells were fixed with 4% paraformaldehyde for 30 min. The cells were then stained with 0.05% crystal violet solution. Cell viability was measured using the Cell Counting Kit-8 (CCK-8) (Beyotime, C0039). Firstly, cell suspension and 100 μl culture medium were added into 96-well plates (4 × 10^3^ GBC-SD cells/well or 2 × 10^3^ NOZ cells/well). Next, at the time points of 0, 24, 48, and 72 h, 10 μl CCK-8 solution was added into each well and incubated for 1 h at 37°C. Finally, the absorbance value for each well was measured at 450 nm wavelength.

### Statistical analyses

GraphPad Prism 8.0 software was used for statistical analysis. Each experiment was performed at least three repetitions, and data are expressed as the mean ± standard deviation (SD). Student’s *t*-test was used to compare the values between two groups when data fit for normal distribution. And one-way ANOVA test was used to compare the values among three or more groups. The *P* < 0.05 was considered to be statistically significant.

## Results

### Expression analysis of DEGs in GBC

**Figure 1:**
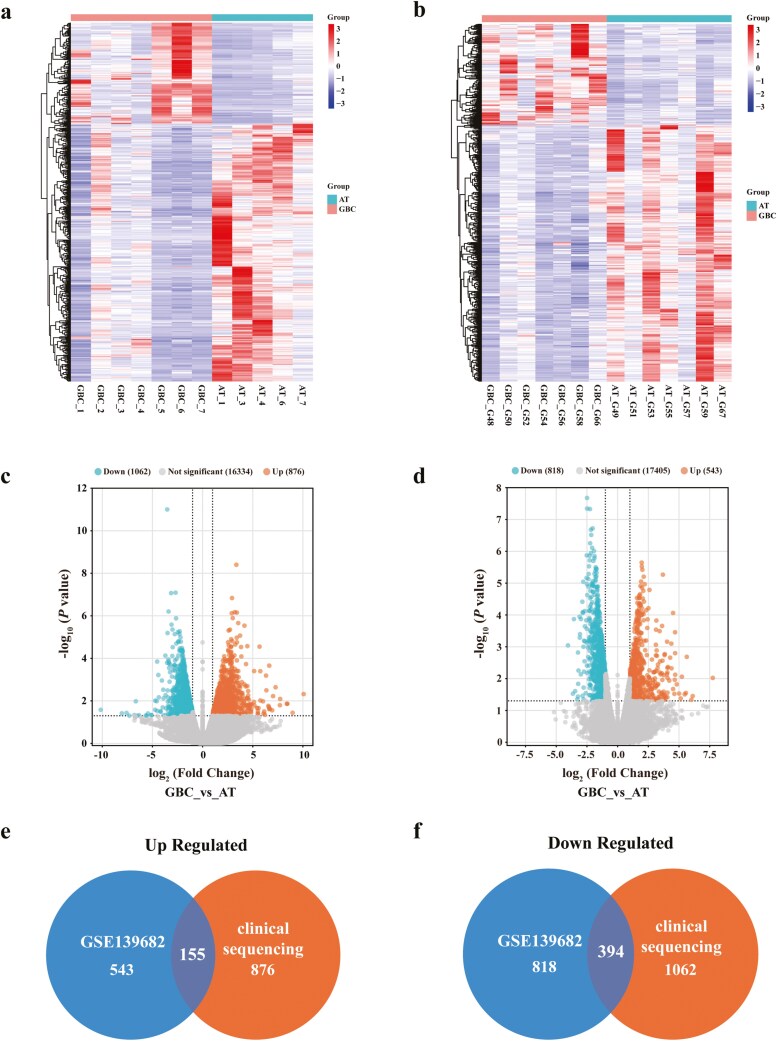
Expression analysis of DEGs in GBC. (a and b) Heatmap analysis of DEGs in clinical sequencing and GSE139682. (c and d) Volcano plots show the DEGs in clinical sequencing and GSE139682. (e and f) Venn diagrams show the intersecting upregulated or downregulated DEGs in the two datasets.

RNA-seq was performed on seven GBC samples and five matched adjacent samples. Gene expression profiles of clinical sequencing and GSE139682 were used to identify DEGs. DESeq was employed to identify DEGs, using significant *P*-value < 0.05 and |log2FoldChange| > 1 as the cutoff criteria (Fig. 1a and b). In clinical sequencing, a total of 1938 DEGs, including 876 upregulated genes and 1062 downregulated genes, were picked out (Fig. 1c). We selected seven pairs of samples’ data in GSE139682 for analysis, because the data volume of the other three pairs is significantly reduced. In GSE139682, a total of 1361 DEGs were identified, containing 543 upregulated genes and 818 downregulated genes (Fig. 1d). Eventually, we took the intersection of the two datasets and obtained 155 upregulated genes (Fig. 1e) and 394 downregulated genes (Fig. 1f), totaling 549 DEGs for further analysis.

### Extracellular matrix remodeling promotes malignant progression of GBC

**Figure 2:**
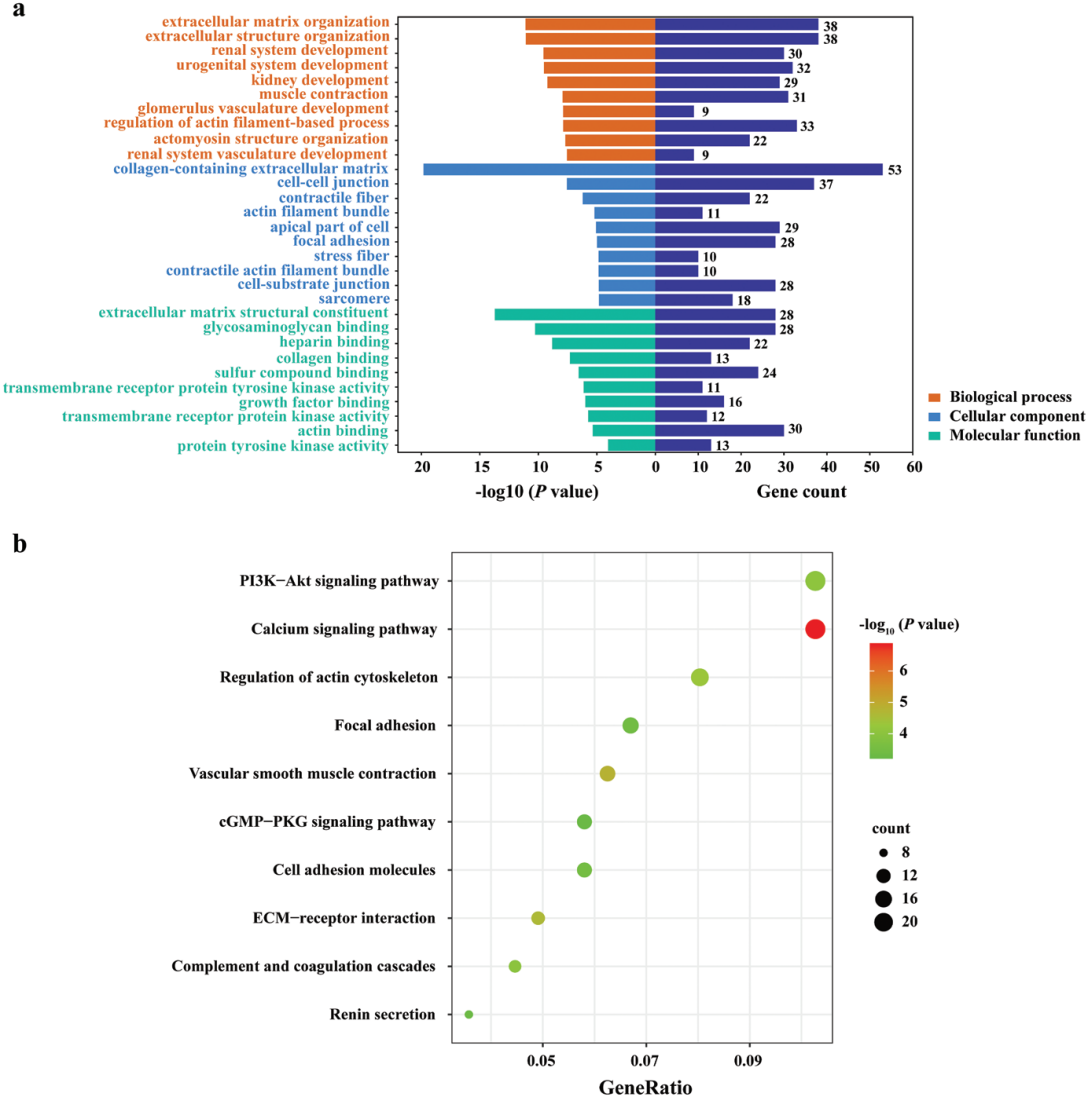
GO and KEGG enrichment analyses of DEGs. (a) The bar plots represent the results of GO function enrichment analysis in DEGs, including biological process (BP), cell component (CC), and molecular function (MF). (b) The bubble plots indicate the results of KEGG pathway enrichment analysis in DEGs.

GO enrichment and KEGG pathway analyses of DEGs were performed using the KOBAS database. For GO enrichment analysis, the DEGs were mainly involved in the following subcategories: extracellular matrix (ECM) organization and extracellular structure organization in the BP, collagen-containing extracellular matrix, cell-cell junction, and contractile fiber in the CC, extracellular matrix structural constituent, glycosaminoglycan binding, and heparin-binding in the MF (Fig. 2a). KEGG pathway analysis indicated that the DEGs were enriched in the PI3K-Akt signaling pathway, calcium signaling pathway, regulation of actin cytoskeleton, focal adhesion, vascular smooth muscle contraction, cGMP-PKG signaling pathway, cell adhesion molecules, ECM-receptor interaction, complement, and coagulation cascades, and renin secretion (Fig. 2b). Based on the above results, we speculate that the changes in extracellular matrix structural components and related signaling pathways may promote the malignant progression of GBC by remodeling the extracellular matrix.

### Abnormal cell division and proliferation are important biological features of GBC

**Figure 3:**
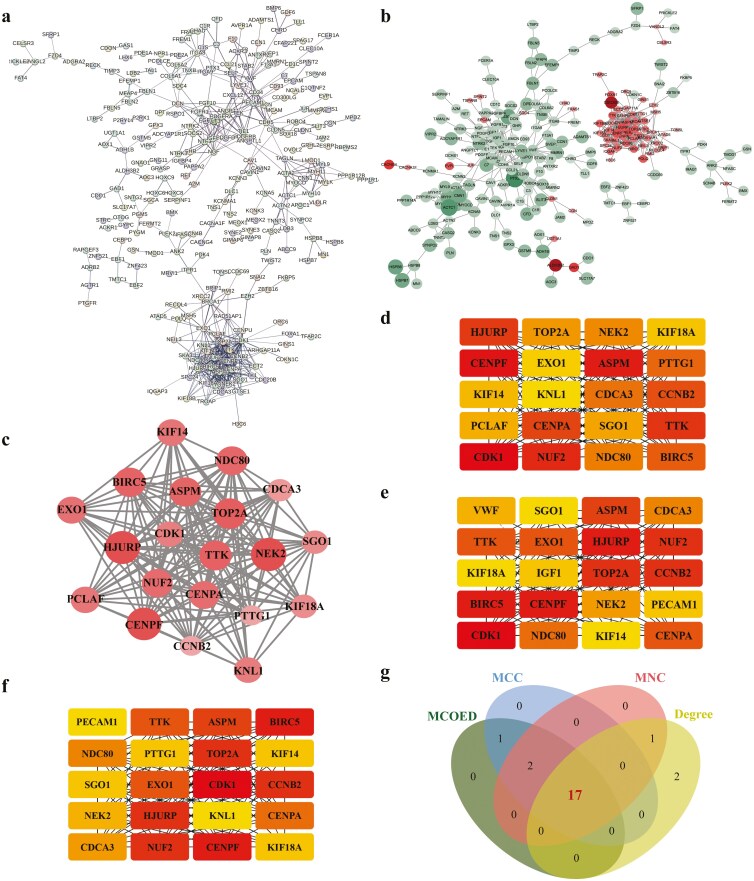
Construction of the PPI network and screening of candidate hub genes. (a) The PPI network was structured by STRING online database. (b) DEGs in the PPI network were labeled using Cytoscape software, using log_2_FoldChange as the standard (including size and color depth, red: upregulated genes; green: downregulated genes). (c) The most significant module was screened using MCODE in the Cytoscape software. (d) The top 20 DEGs obtained according to MCC. (e) The top 20 DEGs obtained according to Degree. (f) The top 20 DEGs obtained according to MNC. (g) Venn diagrams show the 17 intersecting genes in MCC, Degree, MNC, and MCODE.

**Figure 4:**
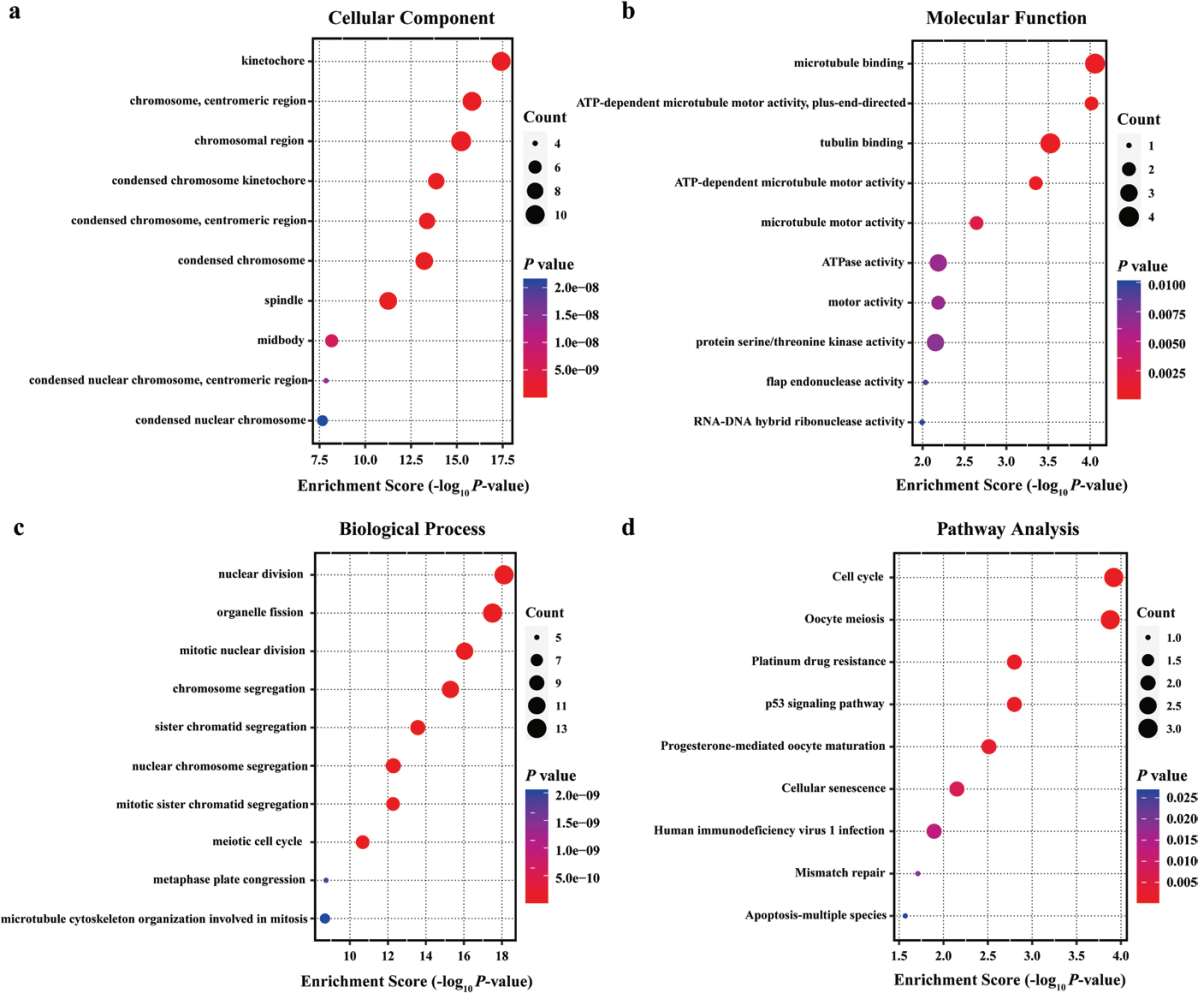
GO and KEGG enrichment analyses of candidate hub genes. (a–c) GO analysis of candidate hub genes. (d) KEGG pathway functional classification and annotation.

We constructed a PPI network using the 549 DEGs by STRING database (Fig. 3a), and the result was visualized using Cytoscape (Fig. 3b). Subsequently, the highest connectivity module, comprising 20 hub genes, was selected by MCODE for further analysis (Fig. 3c). In parallel, the top 20 hub genes were identified using the MCC, Degree, and MNC algorithms of cytoHubba (Fig. 3d–f), respectively. By intersecting the results from the MCC, Degree, MNC, and MCODE modules, we identified 17 candidate hub genes of interest (Fig. 3g). Notably, these genes were all upregulated in the GBC. To explore the characteristics of the 17 hub genes played in GBC, we conducted GO enrichment (Fig. 4a–c) and KEGG pathway analyses (Fig. 4d) once again. The results show that the candidate hub genes were mainly involved in the following subcategories: nuclear division, organelle fission, and mitotic nuclear division in the biological process, kinetochore, chromosomal region, and centromeric region in the cellular components, microtubule binding, ATP-dependent microtubule motor activity, and tubulin binding in the molecular function. And KEGG pathway analysis indicates that the DEGs were enriched in the cell cycle and oocyte meiosis.

### The expression of candidate hub genes in cholangiocarcinoma

**Figure 5:**
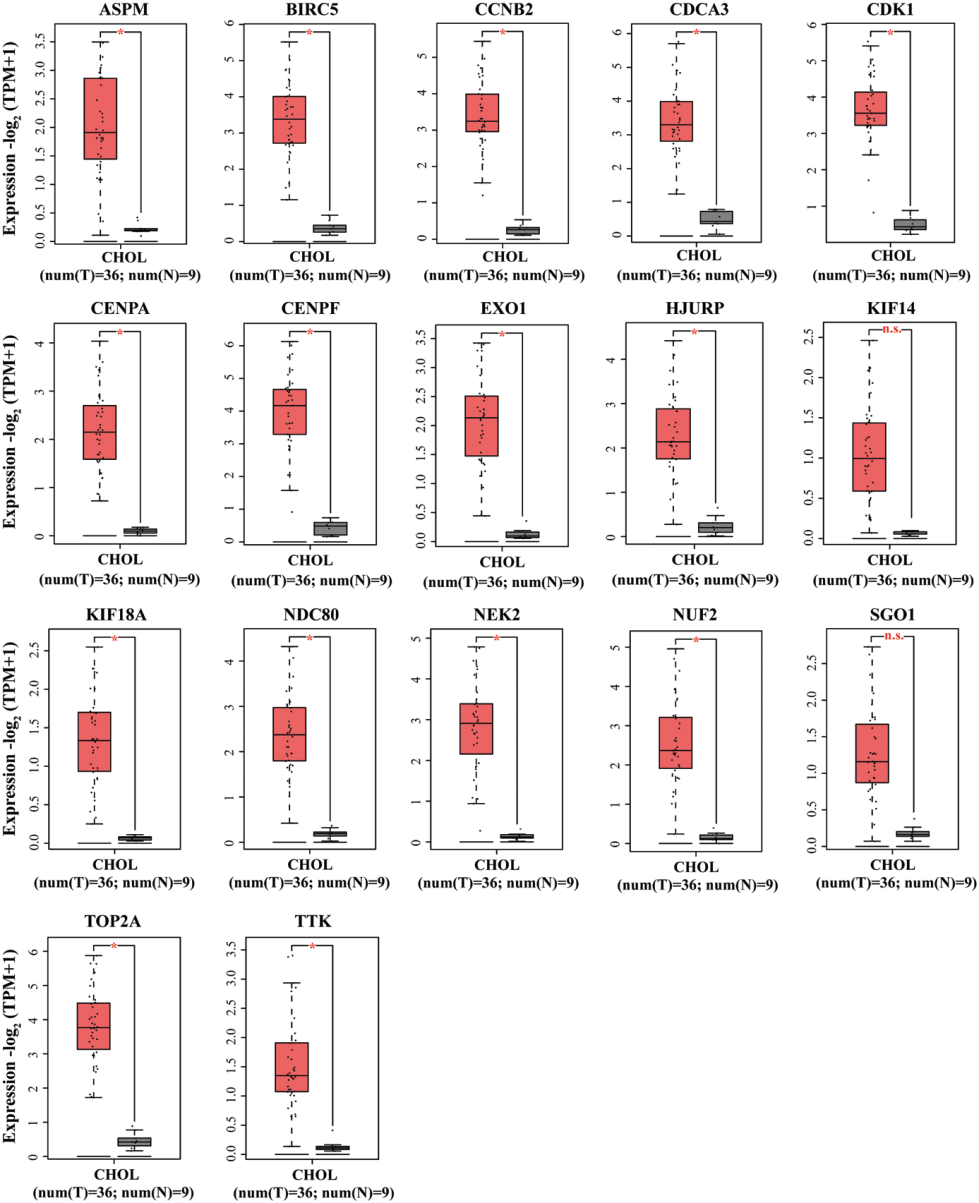
The expression of candidate hub genes in cholangiocarcinoma. Boxplots were displayed to validate the expression of 17 candidate hub genes in cholangiocarcinoma tissues from the GEPIA database. (* *P* < 0.05; n.s., not significant.)

Currently, most databases lack comprehensive sample information for GBC. Given the histological similarities between GBC and cholangiocarcinoma, we analyzed the expression of 17 candidate hub genes in cholangiocarcinoma (CHOL) using the GEPIA database. The results indicated that most candidate hub genes were significantly upregulated in CHOL, consistent with our findings in GBC (Fig. 5).

### NUF2 and NEK2 are hub genes involved in the malignant progression of GBC

**Figure 6:**
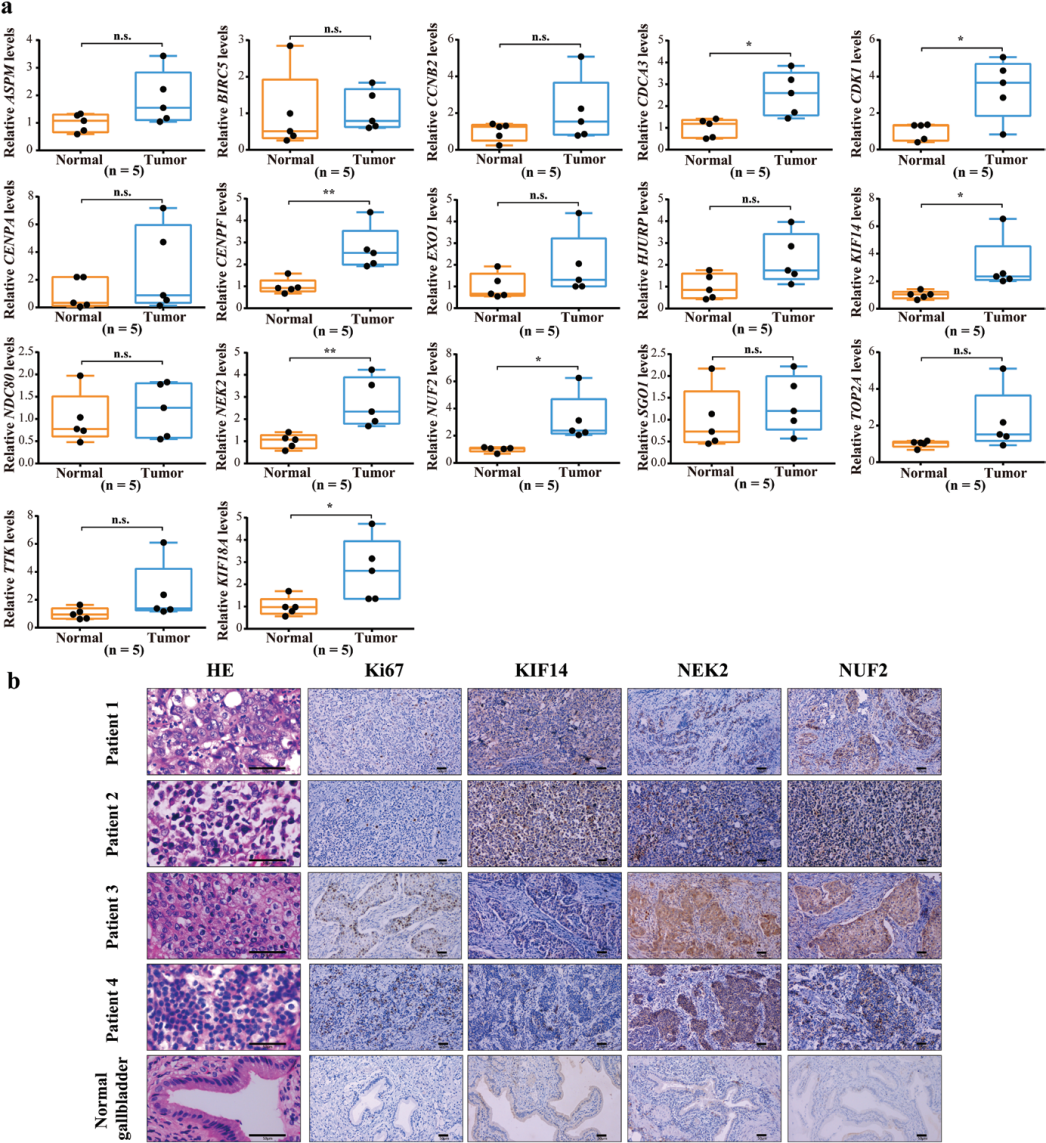
Validation of candidate hub genes by qRT-PCR and IHC. (a) qRT-PCR was used to detect the expression of 17 candidate hub genes in the GBC and adjacent normal tissues (* *P* < 0.05; ** *P* < 0.01). (b) H&E and IHC staining on GBC and normal gallbladder tissue, including NUF2, NEK2, KIF14, and Ki67. Scale bar = 50 μm.

We characterized the clinical relevance of 17 genes in GBC using clinical tissue samples to further refine the selection of hub genes. We measured the expression level of 17 genes by qRT-PCR in five pairs of human GBC tumor samples and matched para-cancerous tissues. The result indicates that the seven genes (*CDCA3*, *CDK1*, *CENPF*, *KIF14*, *NEK2*, *NUF2*, and *KIF18A*) were upregulated in GBC tissue compared with matched para-cancerous tissue (Fig. 6a). Next, we focused on the three genes with the most pronounced differences in expression levels. And we performed H&E and IHC staining on GBC and normal gallbladder tissue, including NUF2, NEK2, KIF14, and Ki67 (Fig. 6b). As shown by IHC, a remarkable increase in NEK2 and NUF2 expression levels was also found in GBC tissues compared to normal tissues.

### NEK2 and NUF2 on GBC cells proliferative function *in vitro*

**Figure 7:**
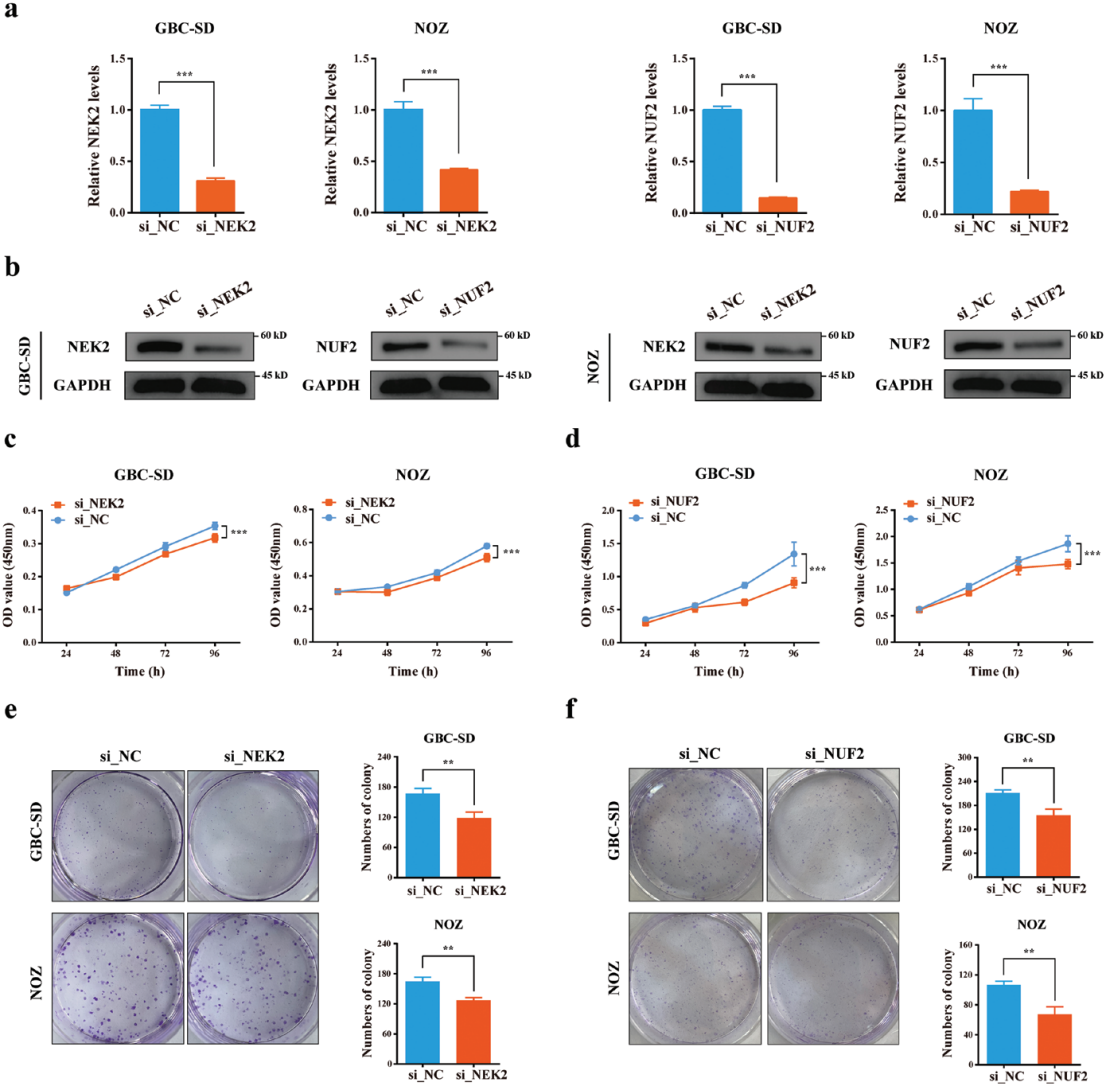
Knockdown of NEK2 and NUF2 inhibited GBC cell proliferation. Validation of knockdown expression efficiency of NEK2 and NUF2 by qRT-PCR (a) and western blot (b). (c) The effect of NEK2 knockdown on the proliferation rate of GBC-SD and NOZ cells was evaluated at 24, 48, 72, and 96 h. (d) The effect of NUF2 knockdown on the proliferation rate of GBC-SD and NOZ cells was evaluated at 24, 48, 72, and 96 h. (e) The effect of NEK2 knockdown on colony formation assay of GBC-SD and NOZ cells was detected after seeding for 10 days. (f) The effect of NUF2 knockdown on colony formation assay of GBC-SD and NOZ cells was detected after seeding for 10 days. (** *P* < 0.01; *** *P* < 0.001.)

To further investigate the functional roles of NEK2 and NUF2 in the malignant progression of GBC, we established GBC cell lines with the knockdown of these genes. We assessed the expression of NEK2 and NUF2 by qRT-PCR and western blotting after processing with siRNA. The results showed that the mRNA and protein expression levels of NEK2 and NUF2 significantly decreased (Fig. 7a and b). Proliferation assays showed that NUF2 and NEK2 knockdown inhibited cell proliferation (Fig. 7c and d). In addition, colony formation assays showed that NUF2 and NEK2 knockdown inhibited cell growth (Fig. 7e and f), which was consistent with the results observed in the organization samples.

## Discussion

GBC is a highly lethal cancer and the most common cancer of the biliary tract. Due to the late onset of clinical symptoms, most GBC patients have progressed to advanced stages at the time of diagnosis and cannot be cured through surgery. Moreover, adjuvant therapies, including radiotherapy, chemotherapy, targeted therapy, and immunotherapy, have not yet achieved significant breakthroughs in GBC and have shown poor efficacy. Owing to these limitations, identifying new therapeutic targets for GBC may provide a better prognosis.

In this study, we identified 549 DEGs with similar expression trends through cross-analysis of clinical sequencing data and the GSE139682 dataset. Next, GO enrichment analysis was performed on these DEGs, and the results showed that the DEGs were significantly enriched in extracellular matrix, containing extracellular structure organization, collagen-containing extracellular matrix, and extracellular matrix structural constituent. KEGG signaling pathway analysis highlighted the importance of the PI3K-Akt signaling pathway and the calcium signaling pathway in GBC. Consistent with the GO analysis, cell adhesion molecules and ECM-receptor interactions were also enriched. It can be inferred that extracellular matrix remodeling plays a critical role during the carcinogenesis of gallbladder tissue. This conclusion is very reasonable, because the extracellular matrix is a highly heterogeneous and dynamically changing non-cellular component in the body, involved in regulating various stages of tumor initiation and progression (15).

Numerous studies have shown that the deposition and cross-linking of large amounts of collagen, fibronectin, and hyaluronic acid (HA) during tumor progression can increase the stiffness of tumor tissue, thereby promoting the malignant phenotype of tumor cells (16). Firstly, increased matrix stiffness can lead to the uncontrolled proliferation of cancer cells. Wu et al. found that rigid ECM stimulates the release of the extracellular vesicles from cancer cells, effectively promoting tumor growth (17). In addition, increased matrix stiffness can disrupt the integrity of vascular structures and promote the formation of tumor blood vessels and branches (18). Meanwhile, the hypoxic environment caused by matrix stiffness in tumor tissue can also stimulate angiogenesis (16,19). Furthermore, the remodeling of the extracellular matrix also plays an irreplaceable role in promoting tumor cell metastasis. Changes in ECM, including an increase in matrix stiffness, can induce epithelial-mesenchymal transition in tumor cells, thereby reducing intercellular adhesion and laying the foundation for cancer cell metastasis (20,21). Prior to cancer cell invasion, the arrangement direction of collagen fibers in the ECM around the tumor gradually changes from circumferential orientation to radial arrangement. This matrix structure, which tends to be arranged radially, is a key regulatory factor for cancer invasion and also provides directional guidance for cancer cell migration (22,23). In addition, metabolic abnormalities in tumor cells are closely linked to abnormal remodeling of the ECM. Liu et al. found that, in stiffer matrices, YAP can act as a mechanical sensor to promote aerobic glycolysis in liver cancer cells (24). Conversely, another investigation implied that a softer matrix is beneficial for the activation of the YAP pathway and glycolytic metabolism (25). Although there have been many studies on the crosstalk between ECM and cancer cell metabolism, their relationship remains incompletely understood. But anyway, no matter how abnormal ECM remodels metabolism, it ultimately promotes cancer cells to produce substantial energy, thereby meeting the energy needs of malignant behaviors such as metastasis. Moreover, the deposition of collagen and HA in ECM mediates the formation of lymphatic vessels in tumor tissue, providing a pathway for tumor cell metastasis (15). In summary, ECM remodeling is involved in the entire process of biological processes of cancer development, including uncontrolled proliferation, metastasis, metabolic reprogramming, and angiogenesis.

The remodeling of the ECM has also caused great difficulties in tumor treatment. β1 integrin is widely expressed in tumor-associated macrophages, tumor-associated fibroblasts, and other tumor stromal cells. It has been detected to be upregulated in various types of cancer tissues and enhances melanoma cells’ resistance to radiotherapy by activating the PI3K/Akt pathway (26). In addition, the stiffened matrix weakens the effectiveness of drug therapy. The stiffened matrix forms a physical barrier against the penetration of drugs, and the study has shown that decreasing the deposited HA in the ECM benefited anti-VEGF therapy in colorectal cancer patients with liver metastasis (27). Moreover, the stiffened matrix can directly act on tumor cells, enhancing the resistance to HER2 inhibitor lapatinib (28). Besides, the hardened ECM induces disorganized tumor blood vessels and loose intercellular connections, which can lead to leakage of chemotherapy drugs (18), thereby having a significant negative effect on drug delivery. This explains why adjuvant therapy is difficult to achieve satisfactory therapeutic effects in GBC.

Given the extensive impact of matrix remodeling on tumors, we are eager to further explore which malignant phenotypes of GBC are most significantly affected by matrix remodeling. To this end, we constructed a PPI network, and 17 candidate hub genes were detected from these DEGs by Cytoscape software. For a more in-depth understanding of these genes, we performed GO and KEGG pathway enrichment analyses again. The GO analysis of BP showed that the candidate hub genes were mainly enriched in nuclear division, chromosome segregation, and cell cycle. The KEGG analysis revealed that the candidate hub genes are mainly related to the cell cycle pathway. From this, we hypothesize that matrix remodeling in GBC mainly affected the cell cycle, thus promoting the malignant progression of gallbladder cancer cells.

Subsequently, qRT-PCR indicated that the expression level of seven genes (*CDCA3*, *CDK1*, *CENPF*, *KIF14*, *NEK2*, *NUF2*, and *KIF18A*) has significantly increased in the GBC tissues compared with the matched para-cancerous tissues. Then, we assessed the protein expression levels of NUF2, KIF14, and NEK2 via IHC, which ranks top 3 in the mRNA expression fold, and the results showed a significant increase of NUF2 and NEK2 in GBC tissue. The high expression of Ki67 indicates a significant enhancement in the proliferation ability of GBC cells. Moreover, the same results were obtained from proliferation assays and colony formation.

NEK2 is a member of the serine/threonine kinases, which is involved in the control of centrosome separation and bipolar spindle formation in mitotic cells and chromatin condensation in meiotic cells. Therefore, the tumor-promoting effect of NEK2 was mainly attributed to its crucial role in promoting the cell cycle. In breast cancer cells, NEK2 knockdown induced cell cycle arrest and led to cell death (29). The study by Zhou et al. found that NEK2 regulates the progression of glioblastoma by modulating the cell cycle (30). NUF2 is an essential component of the NDC80 kinetochore complex, which stabilizes spindle microtubule-kinetochore attachment during cell division, thus ensuring correct chromosome separation during mitosis. Several studies have indicated that NUF2 is upregulated in tumor tissues and regarded as a pro-tumor factor. For instance, reduced expression of NUF2 can arrest melanoma cells in the G0/G1 phase, leading to cell cycle arrest (31). Another study suggested that cell cycle analysis revealed that NUF2 induced G0/G1 cell cycle arrest by inhibiting cyclin B1 expression in breast cancer (32). This is consistent with the observed results that NEK2 and NUF2 promote GBC proliferation in this study.

With ongoing in-depth research, it has been found that they are multi-functional proteins, and their tumor-promoting effect is not limited to what we mentioned above. For example, NEK2 can reprogram glucose metabolism into aerobic glycolysis (33), and NEK2 induces sorafenib resistance in hepatocellular carcinoma tissue by binding to β-catenin (34). In addition, knockout of NEK2 delays tumor progression by significantly reducing the infiltration of tumor-associated macrophages and T cell depletion in multiple myeloma (35). Research has demonstrated that NEK2 primarily exerts its tumor-promoting effects by binding to ATP or interacting with HEC1. Targeting these two biological processes has shown therapeutic potential in several types of cancer. Firstly, SU11652 was the first inhibitor identified to target the ATP-binding site of NEK2 (36). But SU11652 not only inhibits NEK2 but also targets other kinases, such as PLK1. In addition, disrupting the interaction between NEK2 and HEC1 using small-molecule reagents represents another strategy for inhibiting NEK2. For example, INH1 has been shown to effectively inhibit the proliferation of breast cancer cells by blocking the NEK2-HEC1 interaction (37). Currently, a series of INH derivatives, known as TAI, have been developed. Among these, TAI-95 is the only NEK2 inhibitor undergoing phase 1 clinical trials and has demonstrated favorable oral absorption rates and high accumulation in tumor tissues (38). Given the critical role of NEK2 in tumor progression, combining NEK2 inhibitors with first-line anticancer drugs may represent a promising strategy for cancer treatment. Similarly, NUF2 inhibits TFR1 degradation by inhibiting the binding of TFR1 and p62, thereby promoting the progression of CCA through activating downstream p38/MAPK signaling pathways (39). However, there are currently no reported studies utilizing NUF2 as a target for clinical treatment. We believe that NUF2 holds significant promise as a therapeutic target in the future.

Through proteomic analysis of GBC tissues and non-tumor gallbladder tissues, Akhtar et al. found that differentially expressed proteins (DEPs) mainly clustered in three parts, including neutrophil degranulation-related proteins, ECM organization, and cytoskeletal or intermediate filament (40). Meanwhile, they also found that serine proteases released by activated neutrophils such as PRTN3, ELANE, CTSG, and MMP9 were upregulated in GBC tissues. According to reports, they can degrade ECM proteins and promote cancer cell invasion. Therefore, they speculate that there is neutrophil infiltration and degranulation in tumor tissue, leading to ECM protein degradation and promoting migration and invasion of GBC. In our research, we highlighted that remodeling of ECM in GBC tissue can promote the malignant progression of GBC tumors by promoting tumor cell proliferation. This finding enriches the understanding of the mechanisms by which ECM alterations drive GBC development. Simultaneously, we identified the most critical central genes, NUF2 and NEK2, involved in this process and validated their promoting effect on GBC cell proliferation. Given their extensive involvement in the malignant progression of various tumors, they are highly likely to become therapeutic targets of GBC. Troubled by the limited number of patients included, this study has certain limitations. More tissue samples and experimental analysis may needed to validate our hypothesis. In the future, we will continue to analyze the correlation between the hub genes and the malignant phenotype of GBC cells, as well as explore the underlying molecular mechanisms in greater depth.

## Supplementary Material

bgaf019_suppl_Supplementary_Tables_S1-S2

## Data Availability

The RNA-Seq data have been deposited at the Gene Expression Omnibus (GEO) under the accession number GSE276931. All data associated with this study are presented in the article or the Supplementary Data. The data that support the findings of this study are available from the corresponding author upon reasonable request.
