# Supplementary material for: NUF2 and NEK2 promote malignant progression of gallbladder cancer by remodeling the extracellular matrix
Source: Carcinogenesis. 2025 Apr 12;46(2):bgaf019. doi: 10.1093/carcin/bgaf019 (PMC12137899; doi:10.1093/carcin/bgaf019)
Supplement: bgaf019_suppl_Supplementary_Tables_S1-S2 [file bgaf019_suppl_supplementary_tables_s1-s2.docx]

Supplementary Materials for

**NUF2 and NEK2 promote malignant progression of gallbladder cancer by remodeling the extracellular matrix**

Ming Gao^1,3^, Yutong Zhang^2,3^, Peng Ye^3^, Yifan Ren^1,3^, Jun Xu^1, #^.

Author’s primary affiliations

^1^ Hepatobiliary and Pancreatic Surgery and Liver Transplantation Center, First Hospital of Shanxi Medical University, Taiyuan, Shanxi, China.

^2^ Key Laboratory of Cellular Physiology of the Ministry of Education (Shanxi Medical University), Translational Medicine Research Center, Department of Pathology, Shanxi Medical University, Taiyuan, Shanxi, China.

^3^ Faculty of Graduate Studies, Shanxi Medical University, Taiyuan, Shanxi, China

^#^ Correspondence: junxuty@163.com(Jun Xu)

Supplementary Tables

**Table S1.** Primer sequences used in our study.

| Target | Forward primer (5’ - 3’) | | Reverse primer (5’ - 3’) | Application |
| --- | --- | --- | --- | --- |
| ASPM | TTTCAGCCTCTACAAGTCACAACAG | GACCGCCTTCATTCATAGCCAAG | | qRT-PCR |
| BIRC5 | CTCCTTCAGACTTCTCAGTACTC | ATTGCTACTGTGAGCATAACCTAC | | qRT-PCR |
| CCNB2 | GGTACAAGTCCACTCCAAGTTTAGG | GAAGCCAAGAGCAGAGCAGTAATC | | qRT-PCR |
| CDCA3 | ATCTTCTGAATTGGACTTGCCTCTG | GCTGCTTTGCTTCCTCCTTGG | | qRT-PCR |
| CDK1 | TCAGTCTTCAGGATGTGCTTATGC | CCATGTACTGACCAGGAGGGATAG | | qRT-PCR |
| CENPA | TCGGCGGAGACAAGGTTGG | GGCGTCCTCAAAGAGATGAACTAG | | qRT-PCR |
| CENPF | TTCCAGGCGAGTCAGATCAAGG | CCTCCAGGTGGCAGACTTCTC | | qRT-PCR |
| EXO1 | TCATATTCCAGACAAGGCAACAGTG | ATCTCCAAGACCTCCAGACCAAC | | qRT-PCR |
| HJURP | AGAGAACCGTCAGAGGGAGATTG | CCACCAGGACTCGCAGGAC | | qRT-PCR |
| KIF14 | GCTACTGCTGCTACTGGTAGTAATG | TCTGTCTTGGTCTGGGTCATCAC | | qRT-PCR |
| NDC80 | TGTGCCAGTGAGCTTGAGTCC | TCGTCTTTCTTCAGTCGTGGTTTG | | qRT-PCR |
| NEK2 | AAGATTGGAGCAGAAAGAACAGGAG | GGATTACTTGCCAGAGACAGGAAC | | qRT-PCR |
| NUF2 | AACGCCGCACACCAGGAG | TCCATCTGAAAGCTGCTTGAACTC | | qRT-PCR |
| SGO1 | GTCTGAAGTGAGCCTCTGTGAATC | CTGGTGACTGGTCTATCTGAATTGC | | qRT-PCR |
| TOP2A | CAGCAAATGTGGGTTTACGATGAAG | TGTCCGCAGCATTAACTAGAATCTC | | qRT-PCR |
| TTK | CCAGCAGCAACAGCATCAAATAC | GCTTGAACCTCCACTTCCTATCTG | | qRT-PCR |
| KIF18A | CAAACTGCCGAACAACCAAAGC | TTCCACCTGAAGATGAGCAACAAG | | qRT-PCR |
| GAPDH | CAACGACCACTTTGTCAAGCTC | GGTCTACATGGCAACTGTGAGG | | qRT-PCR |

**Table S2.** Sequences of the siRNAs used in our study.

| Name | Sequence (5’ - 3’) |
| --- | --- |
| si-NUF2 | GCATGAAGATGTTAAGCAA |
| si-NEK2 | GUCCUACAAUGAGAAAUCATT |
